# HPV Vaccination among Sexual and Gender Minority Youth Living with or at High-Risk for HIV

**DOI:** 10.3390/vaccines10050815

**Published:** 2022-05-20

**Authors:** Elizabeth Mayfield Arnold, S. Kate Bridges, Cameron Goldbeck, Peter Norwood, Dallas Swendeman, Mary Jane Rotheram-Borus, The Adolescent HIV Medicine Trials Network (ATN) CARES Team

**Affiliations:** 1 Department of Family and Community Medicine, University of Texas Southwestern Medical Center, Dallas, TX 75390, USA; samantha.bridges@childrens.harvard.edu; 2 Center for HIV Identification, Prevention, and Treatment Services, University of California Los Angeles, Los Angeles, CA 90024, USA; cgoldbeck@ucla.edu (C.G.); pnorwood@mednet.ucla.edu (P.N.); dswendeman@mednet.ucla.edu (D.S.); mrotheram@mednet.ucla.edu (M.J.R.-B.)

**Keywords:** HPV, vaccination, HIV, youth, sexual identity, gender identity

## Abstract

Background: Human papillomavirus (HPV) is epidemic among young people, especially those at highest risk of acquiring HPV-related cancers. Methods: Youth aged 14–24 years old (*N* = 1628) were recruited from 13 clinics, community agencies, and social media sites in Los Angeles, California, and New Orleans, Louisiana, that specialized in serving sexual and gender minority youths (SGMY), especially males at risk for HIV. A cross-sectional comparison of sociodemographic and risk histories of HPV vaccinated/unvaccinated youths was conducted using both univariate and multivariate regressions. Results: About half (51.9%) of youth were vaccinated, with similar percentages across states and across genders. Sexual and gender minority youths (SGMY, i.e., gay, bisexual, transgender, and non-heterosexual; 68.8%) and their heterosexual peers (15%) were equally likely to be vaccinated (54%), even though their risk for HPV-related cancers is very different. Vaccinations were higher among younger youth, those not using condoms, youth with greater education, that possessed a primary health care provider, and youth diagnosed with HIV. Vaccinations were lower among youth that were out-of-home due to mental health inpatient hospitalization, drug treatment, homelessness, or incarceration. Conclusions: Special programs are required to target youth experiencing multiple life stressors, especially out-of-home experiences, those with less education, and without the safety net of health insurance or a provider.

## 1. Introduction

Human papillomavirus (HPV) is the most common sexually transmitted infection (STI) worldwide and is associated with cervical cancer and genital warts [1,2,3]. The estimated prevalence rates of HPV for females by the age of 24 years is 44% [4] and 38% for males [5], increasing over time among younger cohorts [6]. With age, lifetime prevalence rates rise to 90% for women and 80% for men [6]. The rates are even higher at a young age for men-who-have-sex-with-men (MSM) [7]. By the ages of 18–26, 69.4% of young MSM have acquired HPV [7] and their rates of anal and genital warts are 17 times higher than for heterosexual peers [8]. Universal uptake of the HPV vaccination could eliminate more than 6.7 million cases of cancer [9]. For example, with only half of young women receiving the HPV vaccine [10,11], there has still been a significant reduction in cervical cancer among young women [12]. 

Because HPV vaccinations were initiated five years later for men than for women [13], vaccination rates are even lower for young men [13,14]. The HPV vaccination data focused on SGMY, that is, gay, bisexual, transgender, and gender non-conforming youth, is scarce [6,14]. Given their heightened risk of cancers, it is fortunate that previous research has found that SGMY initiate HPV vaccinations twice as often as heterosexual adult men, and are four times more likely to have all doses of the HPV vaccine, in contrast to the 50% drop off after the first vaccination among men [15]. The early onset of sexual behaviors and a far heightened risk of acquiring HIV infection among SGMY also increases their rates of HPV [16]. Although rates of HPV vaccination among SGMY are higher than among heterosexual men, rates among both groups are still low and much lower than for women [17,18]. 

All SGMY in the U.S. are at risk for HIV [19], especially gay and bisexual men and those in urban inner-cities where there tend to be higher rates of new HIV cases [20]. Only 39.4% of youth had received the HPV vaccine, and only 59% had ever been tested for HIV [21]. An even more recent study [15], found rates half of the previous year-18.2% based on 2018 data from the same survey. Yet, bisexual youth and lesbian or gay youth had higher rates of vaccination than heterosexuals [15]. This study examines vaccination rates among SGMY, as well as their heterosexual peers, in two urban settings in the United States, Los Angeles, and New Orleans, LA. 

Vaccination rates may also vary by risk behaviors and protective factors, and we examine a range of these factors. This study’s sample was drawn from agencies serving youth with histories of mental health hospitalizations, substance abuse, homelessness, and incarceration, as well as those marginalized for being SGMY. Histories of sexual risk acts, those placing the youth at high risk for HIV and HPV, were particularly common among these youth, as sex was often a means of economic survival. SGMY also lack knowledge about the acquisition and availability of HPV vaccines [22]. There are higher rates of acceptability for the HPV vaccine among those living with HIV [23]. Protective health behaviors, such as obtaining other vaccines and having a primary physician, health insurance, and an annual exam, have also been found to predict at least initiating the vaccine series [11,24]. This study examines not only the relationship of the HPV vaccination uptake to youth ages 14–24 varying by gender and sexual identity, but also the background, sociodemographic, and other health-related factors associated with the uptake of the HPV vaccine. 

## 2. Materials and Methods

### 2.1. Recruitment and Participants

All study procedures followed the guidelines of the Declaration of Helsinki and were approved by the Institutional Review Board of the UCLA (IRB#16-001372; ClinicalTrials.gov NCT03109431). All youths were recruited with voluntary informed consent. Youths aged 14–24 years old present at 13 adolescent clinics and community agencies specializing in services to SGMY and young people who were homeless, subsisted on low income, and lived in neighborhoods with high seroprevalence rates for HIV in New Orleans, Louisiana, and Los Angeles, California (*N* = 1628) were recruited from 2017–2020 [25]. Youths were also recruited via social media and dating apps. Interviewers, who were typically SGMY themselves and who had graduated from college, were placed at each agency, screening youths with interviews when enrolling at the agency. Youths were screened at recruitment to have at least three or more risk factors for HIV, from the following list: being African-American and/or Latina/o/x; gay, bisexual, queer, or another non-heterosexual sexual orientation; transgender or non-binary gender identity; having received substance abuse treatment; having experienced mental health hospitalization; sharing needles for illicit substance abuse; having an HIV seropositive partner in the last year; using drugs other than marijuana in the last four months; or having an STI in the last 12 months. 

### 2.2. Background and Demographic Variables 

After providing written informed consent, participants self-reported their gender identity, sexual orientation, educational history, race, ethnicity, income, employment status, age of sexual debut, and place of residence. Each youth also reported whether they had a health care provider, health insurance, and abstinence/consistent condom use on all sexual encounters. Youth’s histories of incarceration, mental health hospitalization, substance abuse treatment, and homelessness were recorded as occurred (1) or not (0). A variable was created, “out-of-home”, reflecting youth who had experienced any one of these four experiences: homelessness, mental health hospitalization, drug treatment, or incarceration. 

### 2.3. Health-Related Variables 

HPV vaccination status was assessed using the following question: “There is a virus called Human Papillomavirus which can be sexually transmitted. There is a vaccine that can prevent you from getting diseases from this virus. Have you completed the two-shot or three-shot HPV vaccine?” Possible responses included “yes”, “no”, and “I don’t know”, or the option not to answer the question. This question was gated so that youth answering “yes” were queried regarding the number of doses received. HIV status was determined by rapid-HIV testing with CLIA-waived Alere (Waltham, MA, USA) Determine HIV-1/2Ag/Ab Combo finger stick. Those who reported a recent high-risk HIV exposure or had flu-like symptoms received the Cepheid (Sunnyvale, CA, USA) Xpert HIV-1 Qual Assay to test for acute HIV infection that would not be detected by rapid antibody tests. In addition, participants reported on the number of times that they had been diagnosed with an STI (chlamydia, gonorrhea, or syphilis) and their history of sexual protection via either abstinence or consistent condom use with all sexual partners. 

### 2.4. Analytic Methods 

We stratified the sample on self-reported HPV vaccination status and considered those who did not know if they received/completed the vaccination and those who refused to answer the question as missing. We performed bivariate analyses using chi-square (χ^2^) tests to examine possible differences between those vaccinated and not. Multivariate logistic regression model with a binary outcome for HPV vaccination status evaluated factors associated with vaccination [26]. For the variable selection process, we included sociodemographic characteristics, risk, and the protective factors described above, and retained criteria set to significance level α = 0.05. Finally, we compared participants who responded to the HPV vaccination question and those who did not know whether they received/completed the vaccine or refused to answer the question, and performed a sensitivity analysis to examine if the results were similar when classifying those who did not know their HPV vaccination status as not having been vaccinated.

## 3. Results

As noted in Table 1, participants were about 21 years old on average (SD = 2.14), and 80.9% were assigned male sex at birth. Table 1 shows gender and sexual identity separately and in a combined variable indicating that 16.5% of the sample were heterosexual males, 14.7% were females, 55.6% identified gay or bisexual cis-gender males, 8.2% identified as transgender, either female or male; and about 5% were non-binary or gender diverse. Almost half of the participants (49.8%) were Black/African-American, 27% were Latina/o/x, only 16% were White non-Latina/o/x, and 6.5% Asian. More than half (76.4%) had a high school diploma or the equivalent (23% dropped out of school prior to graduation); 45.4% were employed, and about one-quarter (27.2%) were students. Only 28.6% of the sample had an income above the federal poverty level of $1063/month. About three-fourths of the sample (74.1%) had healthcare insurance coverage; 70.7% had a healthcare provider. About half (49.5%) used condoms consistently; about two-thirds (64.3%) did not have any history of STIs, and only about 9% were living with HIV. 

Of the 1628 participants, almost 80% (*N* = 1317) responded to the interviewer’s question regarding HPV vaccination (excluding those who indicated that they did not know if they had received the vaccine and those who did not answer the question) with 51.9% reporting that they had received the vaccine. Participants who responded to the HPV vaccination question differed from non-respondents in that they were more likely to be assigned male sex at birth, white, and unemployed.

Table 2 notes the results of the univariate associations between sociodemographic variables and vaccination status. Females are more likely to be vaccinated than males; with heterosexual males are less likely to be vaccinated than SGMY. Rates of vaccination rise in a linear fashion significantly with education level. Employed young people are more likely to be vaccinated, as are those with a health care provider and insurance. The vaccinated are significantly less likely to use condoms or be abstinent, but equally likely to have an STI. Youth who have been incarcerated, hospitalized for mental health disorder, drug abuse, or are homeless are less likely to be vaccinated. Youth living with HIV are more likely to be vaccinated.

Table 3 shows the results of the multivariate logistic regression model of HPV vaccination on the predictors selected above on whom we had complete data on the variables of interest (*N* = 1099). After adjusting for the other predictors in the model, older youth (adjusted Odds Ratio [aOR]: 0.81, 95% Confidence Interval [CI]: 0.76, 0.87), those who have been out-of-home (aOR = 0.92, 95% CI: 0.87–0.98), and those who are sexually active and not using condoms consistently (aOR: 0.7, 95% CI: 0.54, 0.91) have significantly lower odds of receiving the HPV vaccination. Additionally, those who have a healthcare provider (aOR: 1.74, 95% CI: 131, 2.31), and those who are living with HIV (aOR: 1.75, 95% CI: 1.11, 2.76) had higher odds of being vaccinated for HPV. Moreover, education level was associated with higher odds of being vaccinated. Notably, recruitment site, gender, and being an SGMY are unrelated to HPV vaccination in the multivariate analyses.

## 4. Discussion

This study examined factors influencing HPV vaccination among youth who are at increased risk for or living with HIV. These youth have significant histories of risk behaviors associated with HIV (multiple sexual partners without condoms and STIs), as well as out-of-home placements (that is, homelessness, incarceration, mental health hospitalization, and drug treatment). In contrast to recent studies of HPV vaccinations among young people [21], our sample also reflects sociodemographic factors characterizing those who become HIV infected: 2/3 of the youth are SGMY, and about 80% are African American or Latina/o/x. These life histories place the young people at risk for long-term negative health outcomes, including both HIV and HPV. 

Overall, cisgender women and participants with an “other” (not heterosexual, gay, lesbian, or bisexual) sexual or gender identity have the highest rates of vaccination by gender and sexual identity when examining these factors in the univariate analyses. However, these results do not appear as significant in the multivariate analyses. Given the higher rates of HPV-related cancers among gay, bisexual, and transgender persons, much higher rates of vaccination would be important among these young people. There are now very conflicting data on the uptake of HPV vaccinations among SGMY. Similar to our study, SGMY in a much smaller sample found an HPV vaccination rate of 10% [21]. Yet, other researchers did find higher vaccination rates among SGMY compared to heterosexual peers [15]. 

Most previous research found a need for increased targeting of young men for HPV [24] and that women were more likely to be vaccinated for HPV [27]. This study, which was selected to be a sample of those at high risk for or living with HIV [25], found similar rates of vaccination based on gender and sexual identity in the multivariate analyses. When young people have multiple challenges, it may be that there is less emphasis on getting vaccinated for HPV.

Studies have also shown that HPV vaccination increases for young adult women when healthcare providers recommend the vaccination series [28]. A systematic review showed that providers who strongly endorsed the HPV vaccination, rather than presenting the vaccine as optional or showing their personal discomfort with vaccines, had patients with a higher uptake of HPV vaccinations [29]. These findings speak to the importance of primary care physicians having conversations with youths to clearly outline the long-term benefits of vaccination and dispel any inaccurate myths. 

There is a significant association between HPV vaccination and level of education, which is consistent with a previous study’s findings [30]. Most previous studies focus on how the parents’ education level increases vaccine acceptability, and how doctors tend to focus on parents to approve and facilitate HPV vaccinations [31]. The parents of SGMY may be disadvantaged in not recognizing their children’s increased risk of acquiring HPV, because parents do not know their child’s sexual identity. These parents may also have hesitancy in promoting the HPV vaccine, as parents often misperceive that sexual protection measures may lead to higher rates of sexual encounters and more sexual partners [32,33,34]. 

### Strengths and Limitations

This relatively large sample of SGMY and the sample’s focus on young people with histories of multiple concurrent life challenges (mental health, substance abuse, and homelessness) is a key benefit of this study. Since the data are based on self-reports [21], these reports may not be accurate. However, we do have rapid diagnostic tests of drug use and STIs which indicate youths are reporting many behaviors that could be stigmatizing, and HPV vaccination is not a highly stigmatizing behavior [25,35]. There is a substantial variation in states’ vaccination rates of up to 30% [24]; we do not find this in our study as rates are similar in CA and LA. It may be that among young people at the highest risk for HPV-related cancers, the vaccination rates are similar. Finally, we do not analyze all possible combinations of sexual and gender identity, given small sample sizes in some of these subgroups. 

## 5. Conclusions

Results suggest that despite national efforts to increase the uptake of the vaccine, many youths at high risk for HPV have not received it. SGMY are at the highest risk of acquiring HPV cancers [7,8], but do not appear to be more likely to receive HPV vaccinations. The importance of safety nets (health insurance and providers) is reflected in higher vaccination rates. Institutions that serve other health challenges for young people (e.g., mental health hospitals, substance use treatment, or jails) do not appear to be using the opportunity to vaccinate young people. The higher rates found among youth with more education is often attributed to the youth’s skills or motivation to acquire the vaccine. It may be, however, that schools are a convenient and often used site for vaccinations, and this is a strategy that should be encouraged to facilitate vaccinations. It appears that special programs should target youths with significant life stressors.

## Figures and Tables

**Table 1:** Comparison of sociodemographic and other characteristics by HPV vaccination status (*N* = 1317).

Characteristics	Vaccinated for HPV (*n* = 684, 51.9%)		Not Vaccinated for HPV ^a^ (*n* = 633, 48.1%)		Total (*N* = 1317)	
	***n***	**%**	***n***	**%**	***n***	**%**
Age in years, mean (SD) *	20.83	(2.08)	21.11	(2.19)	20.97	(2.14)
Assessment site						
Los Angeles	361	49.66	366	50.34	727	55.20
New Orleans	323	54.75	267	45.25	590	44.80
Sex assigned at birth **						
Male	530	49.72	536	50.28	1066	80.94
Female	154	61.35	97	38.65	251	19.06
Gender identity *						
Cisgender-female	119	61.34	75	38.66	194	14.73
Cisgender-male	474	49.84	477	50.16	951	72.21
Transgender/gender diverse	91	52.91	81	47.09	172	13.06
Sexual identity *						
Heterosexual	305	54.66	253	45.34	558	42.66
Gay/Lesbian	171	48.86	179	51.14	350	26.76
Bisexual	133	46.18	155	53.82	288	22.02
Other sexual identity	69	61.61	43	38.39	112	8.56
Gender identity and sexual identity **						
Cisgender heterosexual-male	84	38.53	134	61.47	218	16.55
Cisgender gay and bisexual male	390	53.21	343	46.79	733	55.66
Cisgender-female ^b^	119	61.34	75	38.66	194	14.73
Transgender-female ^b^	31	46.97	35	53.03	66	5.01
Transgender-male ^b^	25	58.14	18	41.86	43	3.26
Gender diverse ^b^	35	55.56	28	44.44	63	4.78
Race and Ethnicity						
Black/African American	338	51.52	318	48.48	656	49.81
Latina/o/x	187	51.52	176	48.48	363	27.56
White	116	54.46	97	45.54	213	16.17
Asian/HPI/NativeAmerican/AN/Other	43	50.59	42	49.41	85	6.45
Education level **						
Below high school	124	40.39	183	59.61	307	23.60
High school diploma/equivalent	149	44.48	186	55.52	335	25.75
Some higher education	323	59.59	219	40.41	542	41.66
Completed higher education	88	75.21	29	24.79	117	9.00
Employment **						
Employed	314	53.68	271	46.32	585	45.38
Unemployed	154	43.50	200	56.50	354	27.46
Student	210	60.00	140	40.00	350	27.15
Income above the federal poverty level ($1063.3/month) **						
Yes	223	59.15	154	40.85	377	28.63
No	461	49.04	479	50.96	940	71.37

Note: * *p* < 0.05; ** *p* < 0.001. ^a^ Includes only those answered “no” to the question regarding vaccine status; ^b^ Includes all sexual identities.

**Table 2:** Risk and Protective factors associated with HPV vaccination (*N* = 1317).

Characteristics	Vaccinated for HPV (*n* = 684, 51.9%)	Not Vaccinated for HPV ^a^ (*n* = 633, 48.1%)	Total (*N* = 1317)	Characteristics	Vaccinated for HPV (*n* = 684, 51.9%)	Not Vaccinated for HPV ^a^ (*n* = 633, 48.1%)
	***n***	**%**	***n***	**%**	***n***	**%**
Having a healthcare provider **						
Yes	531	57.16	398	42.84	929	70.70
No	152	39.48	233	60.52	385	29.30
Healthcare insurance coverage **						
Yes	540	55.44	434	44.56	974	74.07
No	144	42.23	197	57.77	341	25.93
Abstinence/Consistent condom use *						
Yes	317	48.18	341	51.82	658	49.96
No	367	55.69	292	44.31	659	50.04
Lifetime history of STIs *						
None	418	49.12	433	50.88	851	64.62
One	149	54.98	122	45.02	271	20.58
Two or more	117	60.00	78	40.00	195	14.81
HIV status *						
Positive	75	64.66	41	35.34	116	8.80
Negative	609	50.71	592	49.29	1201	91.20
Institutional History						
Homelessness **	145	41.0	209	59.0	354	26.9
MH Hospitalization *	168	45.4	176	54.6	370	28.1
Incarceration *	144	45.0	176	55.0	320	24.4
Substance Abuse Treatment *	106	44.4	133	55.6	239	18.1
Any Institutional History **	309	44.8	380	55.2	689	52.3

Note: * *p* < 0.05; ** *p* < 0.001. ^a^ Includes only those answered “no” to the question regarding vaccine status.

**Table 3:** Results of the multivariate analyses examining the rates of HPV vaccinations adjusted for a variety of sociodemographic factors and risk histories.

Variable	aOR	95% CI
Age in Years **	0.97	(0.94, 0.97)
Sex at Birth (Ref: Female)		
Male	0.97	(0.82, 1.13)
Gender ID (Ref: Cis-Female)		
Cis-Male	0.91	(0.75, 1.07)
Transgender/Gender Diverse	0.91	(0.77, 1.05)
Sexual ID (Ref: Bisexual)		
Gay/Lesbian	1.05	(0.97, 1.13)
Heterosexual	1.02	(0.95, 1.09)
Other *	1.15	(1.02, 1.27)
Education Level (Ref: < High School)		
High School *	1.11	(1.02, 1.21)
Some Higher Edu **	1.21	(1.11, 1.30)
Completed Higher Ed **	1.49	(1.35, 1.75)
Employment (Ref: Employed)		
Student	1.02	(0.95, 1.09)
Unemployed	0.98	(0.91, 1.04)
Income (Ref: <1063.33)		
≥1063.33	1.05	(0.98, 1.11)
Healthcare Provider (Ref: No)		
Yes *	1.10	(1.03, 1.17)
Health Insurance (Ref: No)		
Yes	1.05	(0.98, 1.12)
Abstinence/Consistent Condom Use (Ref: No)		
Yes *	0.94	(0.89, 0.99)
Lifetime STIs (Ref: None)		
One	1.02	(0.95, 1.09)
Two or More	1.05	(0.97, 1.14)
Any Out-of-Home History (Ref: No)		
Yes *	0.92	(0.87, 0.98)
HIV Status (Ref: Negative)		
Yes *	1.17	(1.05, 1.28)

Note: * *p* < 0.05; ** *p* < 0.001.

## Data Availability

Data are not available at this time, but a de-identified data set will available through the DASH website in the future as required by the funder.

## References

[1] National Cancer Institute HPV and Cancer. https://www.cancer.gov/about-cancer/causes-prevention/risk/infectious-agents/hpv-and-cancer.

[2] Centers for Disease Control and Prevention Genital HPV Infection—Fact Sheet. https://www.cdc.gov/std/hpv/stdfact-hpv.htm.

[3] Malone M.A., Gower A.L., Reiter P.L., Kiss D.E., McRee A.L. (2021). “What does it matter?” Young sexual minority men discuss their conversations with sexual partners about HPV vaccination. J. Am. Coll. Health.

[4] Dunne E.F., Unger E.R., Sternberg M., McQuillan G., Swan D.C., Patel S.S., Markowitz L.E. (2007). Prevalence of HPV infection among females in the United States. JAMA-J. Am. Med. Assoc..

[5] Gargano J.W., Unger E.R., Liu G., Steinau M., Meites E., Dunne E., Markowitz L.E. (2017). Prevalence of Genital Human Papillomavirus in Males, United States, 2013–2014. J. Infect. Dis..

[6] Chesson H.W., Dunne E.F., Hariri S., Markowitz L.E. (2014). The estimated lifetime probability of acquiring human papillomavirus in the United States. Sex. Transm. Dis..

[7] Meites E., Gorbach P.M., Gratzer B., Panicker G., Steinau M., Collins T., Parrish A., Randel C., McGrath M., Carrasco S. (2016). Monitoring for Human Papillomavirus Vaccine Impact Among Gay, Bisexual, and Other Men Who Have Sex With Men-United States, 2012–2014. J. Infect. Dis.

[8] Hao Z.C., Guo Y.Q., Bowling J., Ledenyi M. (2021). Facilitators and Barriers of HPV Vaccine Acceptance, Initiation, and Completion among LGBTQ Community in the US: A Systematic Review. Int. J. Sex. Health.

[9] Simms K.T., Steinberg J., Caruana M., Smith M.A., Lew J.B., Soerjomataram I., Castle P.E., Bray F., Canfell K. (2019). Impact of scaled up human papillomavirus vaccination and cervical screening and the potential for global elimination of cervical cancer in 181 countries, 2020–2099: A modelling study. Lancet Oncol..

[10] Charlton B.M., Reisner S.L., Agenor M., Gordon A.R., Sarda V., Austin S.B. (2017). Sexual Orientation Disparities in Human Papillomavirus Vaccination in a Longitudinal Cohort of U.S. Males and Females. LGBT Health.

[11] Fontenot H.B., Lee-St John T., Vetters R., Funk D., Grasso C., Mayer K.H. (2016). The Association of Health Seeking Behaviors With Human Papillomavirus Vaccination Status Among High-Risk Urban Youth. Sex. Transm. Dis..

[12] Guo F., Cofie L.E., Berenson A.B. (2018). Cervical Cancer Incidence in Young U.S. Females After Human Papillomavirus Vaccine Introduction. Am. J. Prev. Med..

[13] Walker T.Y., Elam-Evans L.D., Yankey D., Markowitz L.E., Williams C.L., Fredua B., Singleton J.A., Stokley S. (2019). National, Regional, State, and Selected Local Area Vaccination Coverage Among Adolescents Aged 13–17 Years–United States, 2018. MMWR Morb. Mortal. Wkly. Rep..

[14] Lewis R.M., Markowitz L.E., Gargano J.W., Steinau M., Unger E.R. (2018). Prevalence of Genital Human Papillomavirus Among Sexually Experienced Males and Females Aged 14–59 Years, United States, 2013–2014. J. Infect. Dis..

[15] Griffin M., Jaiswal J., Stults C.B. (2021). Human Papillomavirus Vaccination Rates by Gender Identity and Sexual Orientation Among 18–44-Year-Olds in the U.S. Arch. Sex. Behav..

[16] Rodriguez-Alvarez M.I., Gomez-Urquiza J.L., Husein-El Ahmed H., Albendin-Garcia L., Gomez-Salgado J., Canadas-De la Fuente G.A. (2018). Prevalence and Risk Factors of Human Papillomavirus in Male Patients: A Systematic Review and Meta-Analysis. Int. J. Environ. Res. Public Health.

[17] Agenor M., Peitzmeier S.M., Gordon A.R., Charlton B.M., Haneuse S., Potter J., Austin S.B. (2016). Sexual orientation identity disparities in human papillomavirus vaccination initiation and completion among young adult US women and men. Cancer Causes Control..

[18] Beachler D.C., Gonzales F.A., Kobrin S.C., Kreimer A.R. (2016). HPV vaccination initiation after the routine-recommended ages of 11-12 in the United States. Papillomavirus Res..

[19] Centers for Disease Control and Prevention HIV amoung young in the US: Protecting a generation: November 2012: CDC Vital Signs. https://www.cdc.gov/vitalsigns/hivamongyouth/.

[20] Centers for Disease Control and Prevention Ending the HIV Epidemic in the U.S. (EHE): Jurisdictions.

[21] Olusanya O.O., Wigfall L.T., Rossheim M.E., Tomar A., Barry A.E. (2020). Binge drinking, HIV/HPV co-infection risk, and HIV testing: Factors associated with HPV vaccination among young adults in the United States. Prev. Med..

[22] Grandahl M., Neveus T. (2021). Barriers towards HPV Vaccinations for Boys and Young Men: A Narrative Review. Viruses.

[23] Tian T., Wang D., Papamichael C., Yan Z., Guoyao S., Zhanlin Z., Mahan Y., Xiaoqing T., Zheng G., Jianghong D. (2019). HPV vaccination acceptability among men who have sex with men in Urumqi, China. Hum. Vaccin. Immunother..

[24] Fuller K.M., Hinyard L. (2017). Factors Associated with HPV Vaccination in Young Males. J. Community Health.

[25] Rotheram M.J., Fernandez M.I., Lee S.J., Abdalian S.E., Kozina L., Koussa M., Comulada W.S., Klausner J.D., Mayfield Arnold E., Ocasio M.A. (2019). Strategies to treat and prevent HIV in the United States for adolescents and young adults: Protocol for a mixed-methods study. JMIR Res. Protoc..

[26] Harrell F.E. (2001). Regression Modeling Strategies.

[27] McBride K.R., Singh S. (2018). Predictors of Adults’ Knowledge and Awareness of HPV, HPV-Associated Cancers, and the HPV Vaccine: Implications for Health Education. Health Educ. Behav..

[28] Rosenthal S.L., Weiss T.W., Zimet G.D., Ma L., Good M.B., Vichnin M.D. (2011). Predictors of HPV vaccine uptake among women aged 19–26: Importance of a physician’s recommendation. Vaccine.

[29] Gilkey M.B., McRee A.L. (2016). Provider communication about HPV vaccination: A systematic review. Hum. Vaccin. Immunother..

[30] Gorbach P.M., Cook R., Gratzer B., Collins T., Parrish A., Moore J., Kerndt P.R., Crosby R.A., Markowitz L.E., Meites E. (2017). Human Papillomavirus Vaccination Among Young Men Who Have Sex With Men and Transgender Women in 2 US Cities, 2012-2014. Sex. Transm. Dis..

[31] Brewer N.T., Fazekas K.I. (2007). Predictors of HPV vaccine acceptability: A theory-informed, systematic review. Prev. Med..

[32] Newcomb M.E., Feinstein B.A., Matson M., Macapagal K., Mustanski B. (2018). "I Have No Idea What’s Going On Out There:" Parents’ Perspectives on Promoting Sexual Health in Lesbian, Gay, Bisexual, and Transgender Adolescents. Sex. Res. Social Policy.

[33] Polek C., Hardie T. (2017). Changing HPV vaccination rates in bisexual and lesbian women. J. Am. Assoc. Nurse Pract..

[34] Flores D.D., Meanley S.P., Wood S.M., Bauermeister J.A. (2020). Family Characteristics in Sex Communication and Social Support: Implications for Emerging Adult Men Who Have Sex with Men’s PrEP Engagement. Arch. Sex. Behav..

[35] Arnold E.M., Swendeman D., Harris D., Fournier J., Kozina L., Rotheram-Borus M.J. (2019). Stepped Care Intervetnion to Suppress Viral Load Among youth Living with HIV: Study Protocol for a Randomized Controlled Trial. JMIR Res. Protoc..

